# Measurement of glycolysis with heavy water labeling

**DOI:** 10.1016/j.jbc.2026.113082

**Published:** 2026-04-27

**Authors:** Naveed Ziari, Marc K. Hellerstein

**Affiliations:** Department of Nutritional Sciences & Toxicology, University of California at Berkeley, Berkeley, California, USA

**Keywords:** biochemistry, glycolysis, heavy water labeling, isotopic tracing, lactate, mass spectrometry, metabolism

## Abstract

We introduce a method to calculate the contribution of glycolysis to lactate and the sources of cytosolic NADH, using heavy water labeling, mass spectrometric measurement, and combinatorial analysis (mass isotopomer distribution analysis). The number (*n*) of deuterium incorporation sites into C-H bonds during flux through glycolysis *versus* the oxaloacetate/phosphoenolpyruvate-carboxykinase pathway is calculated. The stereospecificity of lactate dehydrogenase reveals the metabolic source of cytosolic NADH used because tricarboxylic acid cycle dehydrogenases have a different spatial orientation (reduce pyruvate from 4R position) than glycolytic GAPDH (4S position, which derives intramolecularly, not from cellular water). We calculate *n* in lactate and validate the model *in vivo* in mouse liver and skeletal muscle and in HepG2 cells. We also compare lactate measurement of glycolysis from [U-^13^C_6_]-glucose to M_3_-lactate. In summary, this heavy water method is operationally simple, provides information about both carbon fluxes and sources of NADH, and is translatable into humans.

Lactate is the end product of glycolysis, but it can also come from carbon flow out of the tricarboxylic acid cycle (TCAC) through the phosphoenolpyruvate-carboxykinase (PEP-CK) reaction from oxaloacetate (OAA). The latter pathway is known as cataplerosis and is the first bottleneck reaction of gluconeogenesis (1). Recent research has identified lactate as a crucial signaling molecule and the primary intercellular link between glycolysis and mitochondrial respiration *in vivo* (2, 3). It is therefore important to have a method that easily and accurately quantifies the activity of pathways that generate this key metabolite. The method presented here applies combinatorial analysis to the deuterium labeling pattern in lactate in the presence of heavy water (^2^H_2_O), to determine both the relative metabolic pathway contributions to lactate carbon and the metabolic sources of cytosolic NADH that reduces pyruvate to lactate.

Mass isotopomer distribution analysis (MIDA) is a technique for measuring the synthesis rate of biopolymers through stable isotope labeling, based on combinatorial probabilities (4, 5). It also holds value beyond that use case through its calculation of various binomial distribution-related parameters such as *n*, the number of potential labeling sites in a polymer (6, 7, 8). For lactate in the presence of ^2^H_2_O, the number of nonacidic covalent C-H bonds that can be labeled with ^2^H differ among the primary metabolic pathways to cytosolic pyruvate and subsequently to lactate—specifically glycolysis (*n* = 1.5) and PEP-CK from OAA (*n* = 4). These pathways differ in the number of C-H bonds that exchange with cellular water during enzymatic reactions in the traversed pathway that involve nonacidic hydride exchange with solvent water. The quantification of deuterium labeling pattern in lactate thereby informs on fractional contributions from each pathway (Fig. 1).

**Figure 1:**
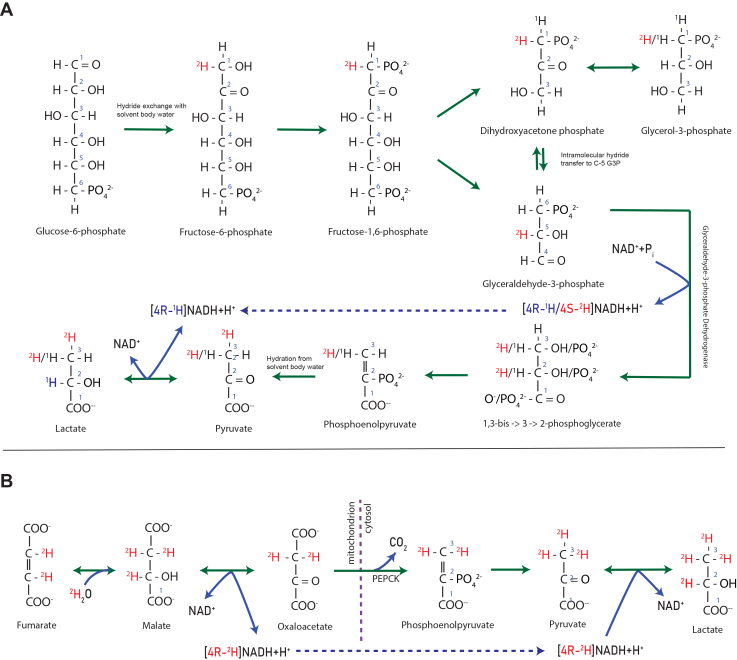
**Deuterium labeling schema in glycolysis and from PEP-CK pathway.***A, n* of glycolysis pathway to pyruvate is 1.5 plus a single label that may be added at the LDH reductive step, the latter being a function of the proportion of NADH generated at GAPDH in glycolysis relative to other dehydrogenases. The ^2^H/^1^H on the methyl group of lactate and pyruvate is 1.5 due to triose-phosphate isomerization, as label from C3 in 1,3-bis-PG (C1 of glucose) is retained, whereas label in C2 of 1,3-bis-PG is not retained in the pyruvate and lactate methyl group. Glycerol input to the triose-phosphate pool is depicted and has the same deuterium labeling signature as glycolysis from glucose (label in C3 of 1,3-bis-PG, retained in pyruvate and lactate). *B, n* of PEP-CK/OAA (TCA cycle) pathway to pyruvate is 3 in the methyl group plus a single label at LDH as a direct proportionality of the fraction of NADH generated in the TCA cycle relative to GAPDH, as noted in (*A*). NADH generated at isocitrate dehydrogenases is not shown but considered in the model as incorporating label from NADH into lactate *via* LDH in the presence of ^2^H_2_O (46). LDH, lactate dehydrogenase; OAA, oxaloacetate; ^2^H_2_O, heavy water; PEP-CK, phosphoenolpyruvate-carboxykinase; TCA, tricarboxylic acid.

This methodology has previously been shown to elucidate other metabolic flux ratios such as metabolic subpathway contributions to gluconeogenesis and to glyceroneogenesis (6, 7, 8).

We describe here a method to measure the relative contributions from glycolysis *versus* PEP-CK/OAA. In addition, a unique aspect of the method described here is that, in addition to determining the metabolic pathways of carbon flow leading to pyruvate and lactate, it can also account for and quantify where the hydrides originate in NADH that is used in redox reactions. This is made possible by the fact that the two hydrides on C-4 of NADH in the cell have differing stereospecificity among dehydrogenase enzymes and differ in exchange with cellular water (9). Specifically, NADH derived from glycolysis, which occurs in the GAPDH reaction, generates NADH hydride in the 4S position, which does not derive from solvent water but is an intramolecular transfer, whereas dehydrogenase reactions in the TCAC generate NADH hydride in the 4R position, which originate from and are equilibrated with solvent water (9). Because lactate dehydrogenase (LDH) reduces pyruvate to lactate from the 4R position of NADH, deuterium is thereby not added to lactate if generated at GAPDH in the presence of ^2^H_2_O. As a result, the method described here not only sheds light on how much pyruvate (lactate) carbon comes from glycolysis but also on the metabolic source of cytosolic NADH used in the LDH reduction step (Fig. 1).

## Results

### Oligomycin treatment increases fractional glycolysis

**Figure 2:**
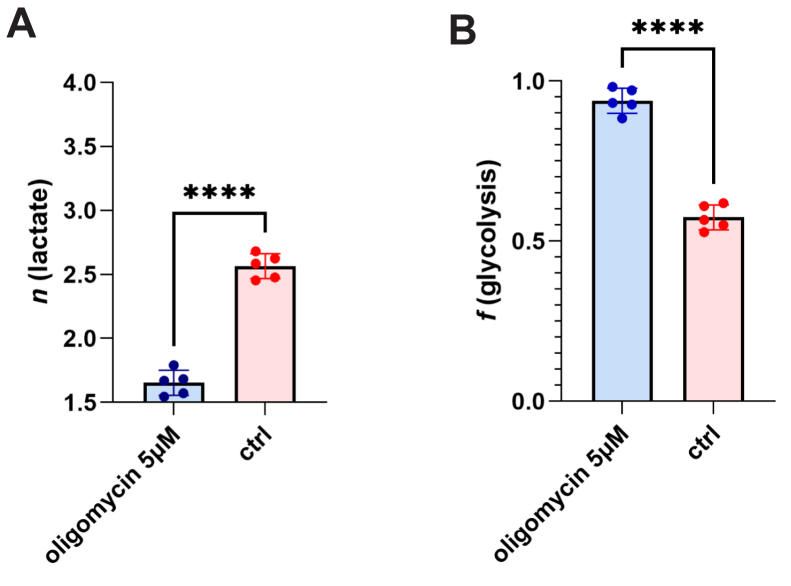
**Oligomycin treatment of cultured HepG2 cells increases fractional glycolysis.***A,* lactate *n* for oligomycin-treated HepG2 cells was 1.654 ± 0.098 (mean ± SD), while for untreated cells 2.56 ± 0.097. *B,* calculated *f*(glycolysis) (%) for oligomycin-treated cells is 94.0 ± 3.9% and 57.4 ± 3.9% for untreated cells (*p* < 0.0001).

The theoretical lower limit for pyruvate *n* is 1.5 and for lactate *n* is also 1.5, in a situation where fractional glycolysis provides 100% of carbon flux and TCAC contribution to NADH at LDH is 0 (Fig. 1*A*). In the basal condition in HepG2 cells, we measured lactate *n* to be close to 2.5 (2.56 ± 0.097) which corresponds to *f*(glycolysis) of 57% (0.576 ± 0.039). When we treated HepG2 cells for 6 h with 5 μM oligomycin, which arrests the electron transport chain by inhibiting ATP synthase and results in an inactive TCAC, as there is no longer a demand for reducing equivalents used in the electron transport chain (10), lactate *n* was significantly reduced to near its theoretical minimum (1.65 ± 0.098, *p* < 0.0001) corresponding to *f*(glycolysis) of approximately 95% (0.940 ± 0.039) (Fig. 2, *A* and *B*).

### [U-^13^C_6_]-glucose labeling of M+3-lactate has comparable readout of *f*(glycolysis) to ^2^H_2_O and provides evidence of pyruvate cycling

**Figure 3:**
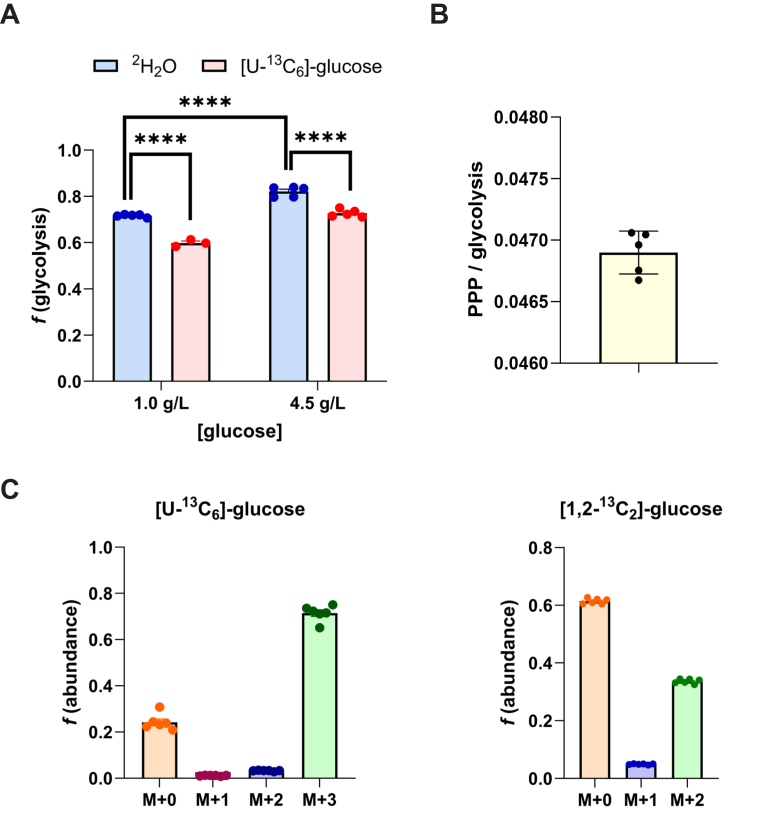
**[U-^13^C_6_]-glucose gives comparable measurement of glycolysis as heavy water and evidence of pyruvate/PEP-CK cycling in cultured HepG2 cells.***A,* fractional glycolysis contribution to hepatic lactate calculated with [U-^13^C_6_]-glucose *versus*^2^H_2_O. Under high glucose conditions (4.5 g/l) in the medium, the [U-^13^C_6_]-glucose method gives 72.7 ± 1.6% and under physiologic glucose (1 g/l) gives 59.3 ± 1.5% contribution to lactate (mean ± SD). This compares to 82.1 ± 2.2% and 71.6 ± 0.6%, respectively, for glycolytic contribution with ^2^H_2_O. The values from ^2^H_2_O from are ∼10% higher than from [U-^13^C_6_]-glucose under both conditions (*p* ≤ 0.0005 in both). The normal glucose condition also resulted in significantly lower fractional glycolysis by 10.5% than high glucose, with both heavy water (*p* = 6.39E-6) and [U-^13^C_6_]-glucose (*p* = 2.59E-5). *B,* [1,2-^13^C_2_] glucose labeling reveals that pentose phosphate pathway flux relative to glycolysis is low (4.7 ± 0.1%). *C,* fractional abundances of lactate mass isotopomers with [U-^13^C_6_]-glucose and [1,2-^13^C_2_] glucose labeling in the studies were summarized in (*A*) and (*B*). PEP-CK, phosphoenolpyruvate-carboxykinase; ^2^H_2_O, heavy water.

Using the same media conditions but replacing the unlabeled glucose with uniformly labeled [U-^13^C_6_]-glucose produces a signal on lactate M+3 isotopomer representing uniformly labeled ^13^C_3_-lactate that must originate from first-pass glycolysis. The fractional abundance of M+3 lactate compared to M+6 glucose, the latter in these conditions being 100%, should therefore be a readout of the first-pass fractional glycolysis contribution to lactate from glucose in the media. We measured 73% (0.73 ± 0.034) (Fig. 3, *A* and *C*). This result is close to that from ^2^H_2_O (82%, Fig. 3*A*), but the ∼10% difference between *f*(glycolysis) calculated with [U-^13^C_6_]-glucose *versus* ^2^H_2_O was worth exploring.

Examination of the lactate M+2 fractional abundance from [U-^13^C_6_]-glucose (0.032 ± 2.0E-3, Fig. 3*C*) represents the fraction of [U-^13^C_6_]-glucose that traverses pyruvate through pyruvate carboxylase to OAA (which equilibrates with fumarate), then through PEPCK to phosphoenolpyruvate (PEP) and back to pyruvate through pyruvate kinase. This pathway has been called pyruvate or PEP cycling (11). Because OAA rapidly equilibrates with fumarate (6, 8, 12), a symmetric molecule, half of the M+3 label in OAA is immediately lost on return to PEP through PEPCK, so true PEP cycling is calculated as double the M+2 signal (*i.e.*, ∼6.4%). Accordingly, PEP cycling explains a substantial fraction of the higher value from ^2^H_2_O than the value of M+3 lactate from [U-^13^C_6_]-glucose.

### [1,2-^13^C_2_] glucose labeling to estimate pentose phosphate pathway flux

When glucose carbons are shunted into the pentose phosphate pathway rather than proceeding through glycolysis, the 6-phosphogluconate (decarboxylating) dehydrogenase reaction strips C-1 (as numbered in glucose). We labeled HepG2 cells with [1,2-^13^C]-glucose and used the model of Katz and Rognstadt (11) to calculate pentose phosphate flux relative to glycolysis. We found this to be less than 5% (0.047 ± 1.74E-4) (Fig. 3*B*). Pentose phosphate flux therefore makes a modest contribution to lactate compared to glycolytic or PEP-CK/OAA (TCAC) inputs, especially under nonmitotic conditions (13).

### Glycogen depletion *in vivo* does not alter lactate *n*

**Figure 4:**
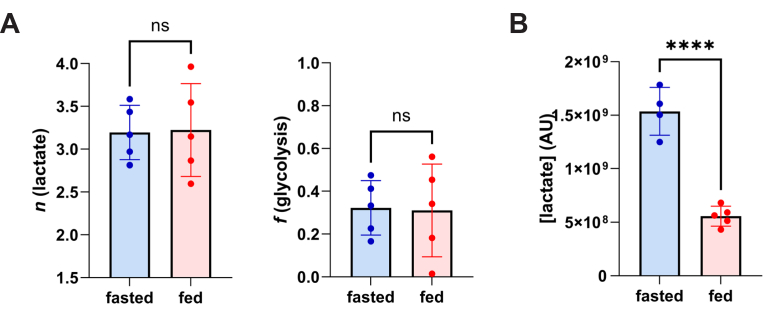
***In vivo* modulation of hepatic glycogen content does not alter *f*(glycolysis) *in vivo* in mice.***A,* 24-h–fasted mice, compared to *ad libitum*–fed, does not alter hepatic lactate *n* (3.19 ± 0.32 *versus* 3.20 ± 0.54, respectively; *p* = 0.919) and hence calculated fractional glycolysis (32.2 ± 12.7 *versus* 31.1 ± 21.6%, respectively; *p* = 0.919). *B,* fasting lowers intracellular lactate levels, measured as M+0 signal intensity, from 15.4 ± 2.2 normalized units to 5.5 ± 0.93 normalized units (*p* = 4.32E-5).

This ^2^H_2_O method includes carbons coming from glycogenolysis in its calculation of *f*(glycolysis), while the [U-^13^C_6_]-glucose method does not but only detects glycolysis from labeled extracellular glucose. We explored the extent of this difference by modulating glycogen levels *in vivo* in mice. Liver samples from *ad libitum*–fed and 24-h–fasted mice were compared. We found no significant difference between the fasted *versus* fed mice (32.2 ± 12.7% *versus* 31.1 ± 21.6% contributions, respectively; *p* = 0.919) (Fig. 4*A*). Notwithstanding its source, fasted mice overall contained less hepatic intracellular lactate, measured as mass spectral intensity (lactate M+0) (Fig. 4*B*), presumably reflecting slower glycolytic metabolism. Administration of a glycogen phosphorylase inhibitor KB228 (14) to HepG2 cells did not significantly change *f*(glycolysis) when measured with the [U-^13^C_6_]-glucose method (59.9 ± 0.82% *versus* 60.9 ± 0.167%, *p =* 0.284) or with ^2^H_2_O (72.5 ± 2.3% *versus* 76.1 ± 1.7%, *p =* 0.010) (Fig. S2). It is worth noting these minute differences are not physiologically meaningful and that *f*(glycolysis) values showed greater variation *in vivo* than *in vitro* in cultured HepG2 cells.

### Fractional glycolysis in nonliver tissue

**Figure 5:**
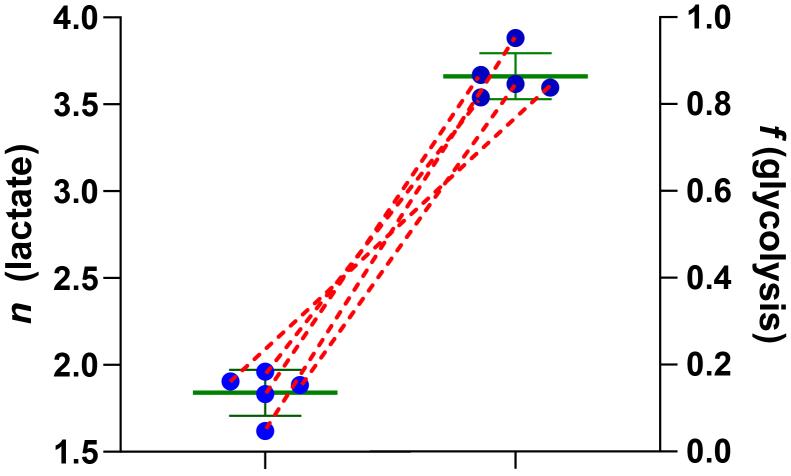
**Muscle glycolysis.** In *ad libitum*–fed mice, lactate *n* in gastrocnemius skeletal muscle is 1.84 ± 0.13, corresponding to a fractional contribution from glycolysis of 86.3 ± 5.3%.

The results presented thus far have been in cultured hepatocytes or liver *in vivo*. Nongluconeogenic cell types also express PEP-CK, as mitochondrial or cytosolic isozymes (15, 16, 17, 18). Skeletal muscle acts as the primary sink for glucose (19) and produces the majority of circulating lactate, which feeds the Cori cycle (20, 21). We measured lactate in *ad libitum*–fed mouse gastrocnemius muscle (Fig. 5). The results reproducibly show that lactate in skeletal muscle primarily comes from glycolysis (lactate *n* = 1.84 ± 0.13, corresponding to fractional glycolysis of 86.3 ± 5.3%).

## Discussion

We present here a method to measure the contribution from glycolysis to pyruvate and lactate *in vivo* and at the same time characterize the dehydrogenase enzymes that generate NADH used in the cytosolic reduction of pyruvate to lactate. While lactate is properly considered an end-product of glycolysis, not all lactate is generated from this pathway, because PEP can also come from PEP-CK (OAA/TCAC), which subsequently gets converted to pyruvate and reduced to lactate (Fig. 1). Knowing the fractional contribution of glycolysis to lactate sheds light on rates of cytosolic glycolysis compared to PEP/gluconeogenesis as well as information to mitochondrial TCAC activity (contribution to cytosolic NADH)—both representing fundamental information in describing intracellular metabolic state.

Regulation of cytosolic redox is of central importance in metabolic regulation (22) but it has not been possible previously to dissect the relative production of NADH from different dehydrogenase enzymes in cells using tracer approaches *in vivo*. We showed here, based on established stereochemical differences between NADH-producing dehydrogenases in glycolysis (GAPDH) *versus* other dehydrogenases (predominately TCAC), that lactate *n* gives a window into where cytosolic NADH is generated in the cell. The reduction of NAD+to NADH varies stereochemically based on the enzyme, which can insert the hydride either into the *pro*-(4S) or the *pro*-(4R) position. GAPDH reacts with the *pro*-(4S) position, while TCAC dehydrogenases and LDH react with the *pro-*(4R) position (Fig. 1; (23). In context of ^2^H_2_O labeling, the *pro*-(4S) position does not exchange with cellular H_2_O but with intramolecular hydrogen (24). A complete list of stereochemical distinctions of all the dehydrogenase enzymes has been articulated and is provided as a list in the Supporting Information (9). While NADH itself does not penetrate the inner mitochondrial membrane, the hydrogens are transferred by malate as a carrier through the malate-aspartate shuttle, generating cytosolic OAA and NADH *via* cytosolic malate dehydrogenase 1 (1, 25), retaining the TCAC stereochemical configuration on NADH. What we have termed here NADH from TCAC reacting at LDH only applies to the reacting hydride, not the intact molecule. Other shuttles such as the glycerophosphate shuttle do not contribute as they are unidirectional from cytosol to mitochondria, while the malate-aspartate shuttle operates in equilibrium at a concentration gradient (26, 27).

Vianello *et al.* found using NMR spectroscopy that during ^2^H_2_O labeling, lowering the oxygen content and hence increasing glycolysis relative to TCAC activity decreases deuterium label in [(2-^2^H)-lactate]/[(3-^2^H)-lactate] (28), where [2-^2^H]- lactate represents label from the LDH reaction. Our model is consistent with this finding but instead uses combinatorial analysis, measured as mass isotopomer ratios, to quantify the number of potential deuterium labeling sites (*n*) on lactate and obtain metabolic flux ratios not only for carbon fluxes into lactate but also for metabolic flux ratios from dehydrogenases into the hydrides of cytosolic NADH.

Oligomycin treatment arrests TCAC flux as the reduced oxygen consumption for ATP synthesis lowers use of and demand for reducing equivalents in the inactive electron transport chain. Our results with oligomycin treatment confirm the theoretical minimum value of *n* in lactate—which stipulates that NADH generated in glycolysis does not label at LDH. The theoretical minimum for *n* is 1.5 when *f*(glycolysis) is 100% and there is no labeling at LDH (Fig. 1*A*). The theoretical maximum for *n* is 4 (Fig. 1*B*), in the absence of glycolysis as source of lactate carbon or NADH labeling, generally consistent with our *in vivo* results in fasted mice (Fig. 4).

Measurement of pyruvate *n* to complement lactate *n* would provide empirical evidence in support of this method’s mathematical implementation of the variable degree of deuterium labeling at LDH. The pyruvate molecule, however, can lose isotopic label to solvent due to keto-enol tautomerism (29), and is insufficient in concentration for accurate analytics. LC-MS and GC-MS measurements of pyruvate consequently yielded inconsistent values (data not shown). We considered that alanine might serve as a superior proxy to pyruvate, being a direct transaminase product while mitigating the constraints of pyruvate. The maximal *n* for alanine is 4.0, while for pyruvate is 3.0 and for lactate is 4.0. We observed for alanine a similar but lower magnitude change in calculated *n* in oligomycin-treated (*n* = 3.10 ± 0.096) *versus* control cells (*n* = 3.52 ± 0.14, *p* < 6.02E-4) than the change in *n* for lactate (Fig. 2, *n* = 1.65 ± 0.063 compared to 2.56 ± 0.054, in oligomycin-treated *versus* controls, *p* < 0.0001). The difference in *n* (Δ *n*) between alanine and lactate in oligomycin-treated cells is 1.45 ± 0.093 and in the basal condition is 0.95 ± 0.058 (*p* < 0.0001, Fig. S1). This finding is consistent with the model that the contribution from glycolysis to lactate alters both label from carbon flux and label from NADH, while the contribution to alanine from glycolysis only alters label from carbon flux.

We validated the model by labeling with [U-^13^C_6_]-glucose to calculate the fraction of lactate that comes from first-pass glycolysis based on the fractional abundance of the M+3-lactate relative to M+6-glucose. This readout is close to but ∼10% less than with the ^2^H_2_O technique. Reducing the media glucose concentration commensurate to normal levels in human physiology (1 g/L) decreased glycolysis but still retained the 10% difference signature between the ^2^H_2_O and [U-^13^C_6_]-glucose labeling techniques (Fig. 2*B*).

We first surmised that this could be due to carbons from glycogenolysis going into glycolysis instead of carbons from labeled glucose in the medium, as HepG2 cells can contain up to 0.8 mg glycogen/mg protein (30). To investigate this further, we modulated glycogen content *in vivo* by calculating fractional glycolysis from ^2^H_2_O labeling experiments *in vivo* in *ad libitum*–fed *versus* 24-h–fasted mice and found (12) no significant difference (Fig. 4*A*). There is greater variation *in vivo* among biological replicates within each condition, so we additionally tested within the HepG2 cell line and performed an experiment with a glycogen phosphorylase inhibitor KP-228 (14) and found no biologically significant difference in *f*(glycolysis) from ^2^H_2_O and [U-^13^C_6_]-glucose labeling (Fig. S2).

We also administered [1,2 – ^13^C_2_] glucose to estimate pentose phosphate pathway flux and found it to be less than 5% relative to glycolysis. Multiplying the results from the two ^13^C experiments (glycolysis contribution and pentose-phosphate relative to glycolysis contributions) demonstrate that a rather insignificant amount of lactate derives from the pentose phosphate pathway. The oxidative portion of this pathway expires the label on C-1 as CO_2_, further dampening the M+3 isotopomer readout in lactate. With ^2^H_2_O labeling, the effect of transaldolase exchange on previously described methods for measuring gluconeogenesis into C-5 of glucose has been discussed in the literature (8, 31, 32). This concern, however, is not implicated in the deuterium labeling pattern of the glycolysis pathway that we measure here, as deuterium in position C5 of glucose (C2 of 1,3-bis-PG) is not retained in the methyl carbon of pyruvate and lactate (Fig. 1*A*). Further experimentation revealed the main source of discrepancy to be loss of [U-^13^C_6_]-glucose label from pyruvate (PEP-CK) cycling. The futile metabolic cycle traversing pyruvate carboxylase, OAA, PEP-CK, and pyruvate kinase has been characterized in the liver (33). [U-^13^C_6_]-glucose labeling here produced evidence of this cycle’s activity through the presence of a M+2 signal on lactate, resulting from traversal of pyruvate carboxylase and PEP-CK, rather than first-pass glycolysis to lactate after pyruvate kinase. OAA equilibrates rapidly with fumarate (6, 12, 34), a symmetric molecule, resulting in the conversion of M+3 signal into M+2 due to CO_2_ release at PEP-CK, even without full traversal of the TCAC. Results with [U-^13^C_6_]-glucose demonstrated that 6.4% of pyruvate originating from glycolysis goes through pyruvate cycling which converts half its flux from M+3-lactate to M+2-lactate.

Lastly, it is necessary to consider glycerol entry into the triose-phosphate (P) pool *via* glycerokinase. The deuterium labeling pattern on lactate is the same in first-pass glycolysis from exogenous glucose or from free glycerol entry into triose-P (Fig. 1*A*). However, with [U-^13^C_6_]-glucose, unlabeled glycerol entry into the triose-P pool will dilute the isotopic label and consequently the *f*(glycolysis) readout. This once again highlights the fundamental difference between the two methods: ^2^H_2_O measures pathway contribution through glycolysis while carbon-13 methods measure specific carbon sources such as extracellular glucose. *In vivo*, a small fraction (up to 13%) of circulating lactate derives from glycerol (35), an effect attributable to peripheral tissues such as skeletal muscle that express glycerokinase but are not gluconeogenic (36, 37). Of note, we demonstrate the utility of this ^2^H_2_O labeling method in other tissues by measuring fractional glycolysis in skeletal muscle (Fig. 5).

This method is robust analytically because it requires the enrichment of only mass isotopomer M+1. Other metabolic flux ratio methods premised on often MIDA use the EM2/EM1 (EM = enrichment of mass isotopomer 1, 2, ...) ratio because it is immune to dilution by unlabeled species (4). That is not an issue here because turnover of lactate in the cell is fast relative to the duration of ^2^H_2_O labeling, meaning that all the lactate measured is newly synthesized after 24 h of exposure to isotopic label (*i.e., f* is 100%). This makes human studies much more feasible and accurate, because body water enrichments in human trials typically do not go above 1 to 2% (4, 38) which gives a low EM2 signal in small molecules like lactate. For the *in vitro* experiments, we used alanine- and pyruvate-free media to avoid having any unlabeled pyruvate or alanine from media. We realize that, *in vivo,* an unknown amount of alanine could come from protein catabolism, although most of this is expected to equilibrate rapidly through the highly active alanine transaminase with pyruvate pools.

Another distinction with regards to analytical capability is that while the extraction protocol and mass spectrometry setup described here is generalizable to metabolomics and the detection of other small molecules, lactate is the largest peak on the chromatogram by LC-MS with the samples used in this study. Lactate typically has one of the highest signal-to-noise ratios in an untargeted metabolomics approach.

This method is an easy and accurate way to measure fractional glycolysis and demonstrates that the labeling pattern of lactate is an important tool to investigate physiologic and pathological states. ^2^H_2_O labeling offers a solution to many of the problems extant in [U-^13^C_6_]-glucose labeling, which may include logistics and costs with human cohorts, and nonlinear amplification from any small deviation in the model, as noted with the observation of pyruvate cycling in our [U-^13^C_6_]-glucose labeling studies. ^2^H_2_O labeling overcomes many of the drawbacks of carbon-13 labeling and renders *in vivo* studies easier to conduct.

In closing, an example worth noting here is that cancer is increasingly recognized to be a metabolic disease (39), whereby mitosis supersedes the demand for oxygen delivery from the vasculature and preferentially upregulates glycolysis (40). Having a simple method to calculate the extent of this metabolic signature could be of importance in cancer research and prognosis. For the study of biology in the main, metabolic fluxes are indeed the paramount phenotype, and methods to easily measure key metabolic fluxes into lactate and pyruvate may serve as a valuable tool for researchers and clinicians.

## Experimental procedures

### Cell culture

HepG2 cells were obtained as frozen vials, tested for authenticity and absence of *mycoplasma*, from the University of California, Berkeley cell culture facility and subsequently expanded. For the ^2^H_2_O experiments, individual 10 cm petri dishes (Corning #430167) were plated with 6.4e6 cells with 16 ml of media. For the carbon-13 experiments, 360,000 cells were seeded onto 6-well plates with 3 ml of media. All cells were incubated for 48 h after plating. The media are comprising 500 ml Dulbecco's modified Eagle's medium (DMEM, Gibco 11965092), 50 ml of fetal bovine serum (Gibco A3160702), and 5 ml of penicillin/streptomycin (Gibco 15140122).

A 24-h labeling duration for ^2^H_2_O (16-h for [U-^13^C_6_]-glucose) commenced after the 48-h incubation period by altering the DMEM in the media formulation described above to one without glucose and without pyruvate (Gibco 11966025). Pyruvate is omitted from cell culture conditions to ensure that all the pyruvate (and thus lactate) comes from either glycolysis or PEPCK. For the carbon-13 experiments, 2.325*g* of [U-^13^C_6_] glucose (Cambridge Isotope Laboratories CLM-1396-1) were added. For the ^2^H_2_O experiments, 2.25*g* of (unlabeled) glucose were added to the 500 ml DMEM bottle, and 44 ml of pH-calibrated ^2^H_2_O solution comprising 1:9 parts 10X PBS: 100% deuterium oxide (Sigma 269786).

Oligomycin (Sigma Aldrich 75351-5 mg) was added as a 1:1000 dilution to 5 μM from a 5 mM 100% dimethyl sulfoxide (DMSO, Molecular Probe D12345) solution stored at −20 °C and treated for 5 h. A commensurate amount of DMSO was added for control conditions. Glycogen phosphorylase inhibitor KB-228 treatment dissolved in water at 3 mM and added as a 1:1000 dilution to 3 μM and treated for 8 h.

After the labeling period, 800 μl of the media from the ^2^H_2_O experiments were aliquoted for water enrichment analysis (see section “Measurement of 2H2O” enrichment). Media was then removed, and cells were washed 3 times with PBS. For the ^2^H_2_O experiments, the PBS wash included 8% (pH-calibrated) ^2^H_2_O to keep water enrichment consistent while the cells are still metabolically active. Metabolism was then quenched with 1 ml of ice cold methanol and then scraped into an Eppendorf tube and then stored at −80 °C.

### Animal experiments

Animal experiments were conducted according to the animal use protocol, approved by the University of California, Berkeley, Animal Care and Use Committee. C57BL/6J male mice aged 8 to 12 weeks were acquired from the Jackson laboratory in Bar Harbor, MA (Strain # 000664). Prior to specifying any experimental perturbation, mice were fed standard chow diet and water *ad libitum*. Mice were fasted for 24 h by the removal of food from their cage. Four hours prior to sacrifice, mice were given a bolus intraperitoneal injection of 100% ^2^H_2_O (Sigma Aldrich 269786) at 35 ul/g body weight, then switched from drinking water to 8% ^2^H_2_O. Immediately prior to sacrifice, 400 μl of blood was drawn by cardiac puncture under isoflurane anesthesia. After sacrifice by cervical dislocation, liver was taken and stored in methanol. In the fed group, the gastrocnemius was also dissected. Samples were frozen at −80 °C.

### Extraction

For cells, the methanol evaporated in a speedvac system until the sample became dry. One milliliter of extraction buffer, a mixture of 2:2:1 LC-MS grade acetonitrile:methanol:water was added and each vial underwent a cycle repeated 3 times of (1) 1 min vortexing (2) 1 min liquid nitrogen exposure, and (3) 10 min of sonication in room temperature water. Later, the samples were left to incubate for 2 h at −20 °C. The samples were then centrifuged for 10 min at 4 °C at 14,000 rpm. The supernatant was then transferred to a new Eppendorf tube and then dried in a speedvac. Simultaneously, a bichinconinic acid protein mass quantification assay was performed on the sediment. Once dry, a mixture of 2:1 LC-MS grade acetonitrile:water was added at a volume of 1 μl/3.2 μg of protein. The samples were then twice 1) sonicated in room temperature water for 5 min and 2) vortexed for 30 s, and subsequently stored in 4 degrees for 2 h (the duration of storage at −20 and 4 degrees could vary from 1 h to overnight). The samples were then centrifuged at 14,000 rpm at 4 °C for 10 min, and the supernatant was transferred to an LC-MS vial and stored at −80 °C.

For mouse tissues, extraction buffer (2:2:1 LC-MS grade acetonitrile:methanol:water) was added at 40 μl/mg tissue and homogenized for 4 min at a frequency of 30 s^−1^. The sample was then incubated for 2 h at −20 °C and centrifuged at 14,000 rpm at 4 °C for 10 min. The supernatant was aliquoted into LC-MS vials, and stored at −80 °C.

### Mass spectrometry

Ultra-high performance LC/MS was performed with a ThermoFisher Vanquish liquid chromatography system coupled with a ThermoFisher Q-Exactive Plus orbitrap mass spectrometer. Hydrophilic interaction liquid chromatography (HILIC) was conducted with a HILICON iHILIC-(P) classic guard column (20 mm × 2.1 mm, 5 μm) connected to a HILICON iHILIC-(P) classic HILIC column (100 mm × 2.1 mm, 5 μm). Mobile-phase solvents were composed of A = 20 mM ammonium bicarbonate, 0.1% ammonium hydroxide, and 2.5 μM medronic acid in water:acetonitrile (95:5) and B = 2.5 μM medronic acid in acetonitrile:water (95:5). The column compartment was maintained at 45 °C for all experiments. The following linear gradient was applied at a flow rate of 250 μl min^-1^: 0 to 1 min: 90% B, 1 to 12 min: 90 to 35% B, 12 to 12.5 min: 35 to 25% B, 12.5 to 14.5 min: 25% B. The column was re-equilibrated with 20 column volumes of 90% B. The injection volume was 2.56 μl.

Data were collected with the following settings: spray voltage, −3 kV; sheath gas, 35; auxiliary gas, 10; sweep gas, 1; ion transfer tube temperature, 250 °C; vaporizer temperature, 300 °C, mass range, 70 to 1000 Da, and two narrow-mass ranges, 270 to 400 Da and 550 to 700 Da; resolution, 240,000 (MS1), 30,000 (MS/MS); maximum injection time, 500 ms; isolation window, 1.5 Da; polarity, negative.

The peak for lactate anion ([C_3_H_6_O_3_ – H]^-^) was extracted with 0.09 ppm error using ThermoFisher FreeStyle software (version 1.6). The peak height for each nominal mass isotopomer M+0, M+1, and M+2 is revealed by the mass spectrum for the center of the lactate peak. Peaks heights were normalized to obtain fractional abundances for M+0, M+1, and M+2 mass isotopomers. Retention times of peaks were determined with injection of unlabeled lactate (Sigma-Aldrich L7022)) standard. During method development, it was found that peak heights reliably provide the same analytical performance as full integration of peaks using selected ion monitoring with a mass window of 87 to 93 atomic mass units (data not shown), but operationally more efficient. The mass spectrometric method described above is adapted from (41). It is also possible to use a GC-MS system to measure lactate (42, 43).

### Measurement of ^2^H_2_O enrichment

Prior to harvesting cells, 800 μl of media was sampled to measure the isotopic enrichment of the media. For mouse experiments, 400 μl of blood was collected upon sacrificing. One hundred twenty microliters of blood or media was distilled. Water enrichment was calculated using the acetone method described in Yang et al. (44). The distilled water was then set to incubate overnight in 2 μl of 10N NaOH and 4 μl of acetone. The next day, 300 μl of hexane were added and the sample was vortexed, precipitating the acetone into the organic hexane phase. Two hundred microliters of the organic phase was drawn into a GC vial and sealed tightly.

Acetone was measured on a GC-MS system in electron ionization mode with a DB-225 column (Agilent #122-2962) and scanned for ions *m/z* 57, 58, and 59 for the M+0, M+1, and M+2 mass isotopomers. Regression on a standard curve using water enrichment at different levels will show a relationship between fractional M+1 abundance and the water deuterium enrichment *p*, allowing calculation of *p* with the independent variable being M+1 for the sample.

### Calculation of the number of exchangeable covalent C-H bonds (*n*)

Lactate *n* is calculated by generating MIDA tables for lactate (C_3_H_6_O_3_) using PyMIDA (45) for any two integer-valued *n* (*e.g. n* = 2 and *n* = 3). A relationship between lactate EM1 (enrichment of mass isotopomer M1 in undiluted newly synthesized lactate) and *n* is thereby established for any given *p.* EM1 is calculated by subtracting baseline natural abundance M+1 (0.0337) from the M+1 signal. An equation for a line was created on Microsoft Excel, whereby lactate EM1 is the independent variable (x) and *n* as the dependent variable (y). This function then output *n* of lactate as a function of EM1. The assumption here is that lactate is rapidly turned over and therefore at maximal EM1 after several hours of exposure to ^2^H_2_O. Examples of data and tables going through this procedure are shown in the Supporting Information. Alanine *n* was calculated in similar fashion but instead using EM2/EM1 ratio instead of EM1 (45).

### Model

Pyruvate derived from glycolysis confers *n* = 1.5 units of deuterium label on the methyl group (Fig. 1*A*), whereas PEP-CK (OAA)-derived pyruvate labels the entire methyl group with *n* = 3 units of deuterium label (Fig. 1*B*). The NADH generated in first-pass glycolysis at GAPDH does not exchange into the 4R position but enters into the 4S position, and the hydride transferred comes intramolecularly from glucose, not cellular water. Because hydrogen is incorporated by LDH from the 4R position of NADH into C-2 of lactate (9), first-pass glycolysis therefore does not label lactate C-2 through LDH. In contrast, NADH generated in the TCAC or from most other dehydrogenases (9), transfers hydride from the 4R position, and originates from cellular water. Accordingly, the probability of reducing pyruvate with cellular water-derived ^1^H/^2^H in cytosolic NADH is inversely proportional to the fractional contribution from first-pass glycolytic flux vs the TCAC. This is articulated in the equation below showing that *n* of lactate is the weighted sum of *n* for each pathway (*i.e. n* from carbon flux plus *n* from NADH at LDH):

$$n_{lactate}=\alpha \left[1.5+\left(1-\alpha \right)\right]+\left(1-\alpha \right)\left[3+\left(1-\alpha \right)\right]$$

where α denotes fractional contribution from glycolysis and (1 − α) is the proportion coming from TCAC. For glycolysis, *n* is therefore 1.5 plus the (1 − α) probability of labeling at LDH. For the PEP-CK pathway, *n* is 3 (the entire methyl group being labeled) plus the (1 − α) probability of labeling at LDH. It should be noted that the primary underlying assumption in this model is that the fraction of NADH generated in glycolysis at GAPDH is the same fraction of NADH that reacts at LDH. A singular measurement of lactate necessitates such an (experimentally validated) assumption in an otherwise underdetermined system. Solving for α (*i.e.* fractional contribution from glycolysis) yields:

$$f\left(glycolysis\right)=\frac{4-n}{2.5}$$

## Statistical analysis and data visualization

Statistical Analysis was performed in Microsoft Excel and GraphPad Prism v10 (https://www.adobe.com/products/illustrator.html). Significance was determined using a two-tailed Student’s *t* test. Figures were made on GraphPad Prism v10 and Adobe Illustrator 30.0. Errors are represented as SDs. All data points on the graph are individual biological replicates—separate animals or petri dishes.

## Data availability

This study, its conception, and experiments were conducted by N. Z. under the guidance of M. H. The raw data and calculations for Figure 4 are in Supporting Information. Requests for further information, resources, and raw data should be directed to and will be fulfilled by the lead contact, Naveed Ziari (naveedziari@berkeley.edu) or may be addressed to the PI (march@berkeley.edu).

## Supporting information

This article contains supporting information.

## Conflict of interest

The authors declare that they have no conflicts of interest with the contents of this article.
