# Supplementary material for: Measurement of glycolysis with heavy water labeling
Source: J Biol Chem. 2026 Apr 27;302(6):113082. doi: 10.1016/j.jbc.2026.113082 (PMC13241725; doi:10.1016/j.jbc.2026.113082)
Supplement: Supplementary material [file mmc1.docx]

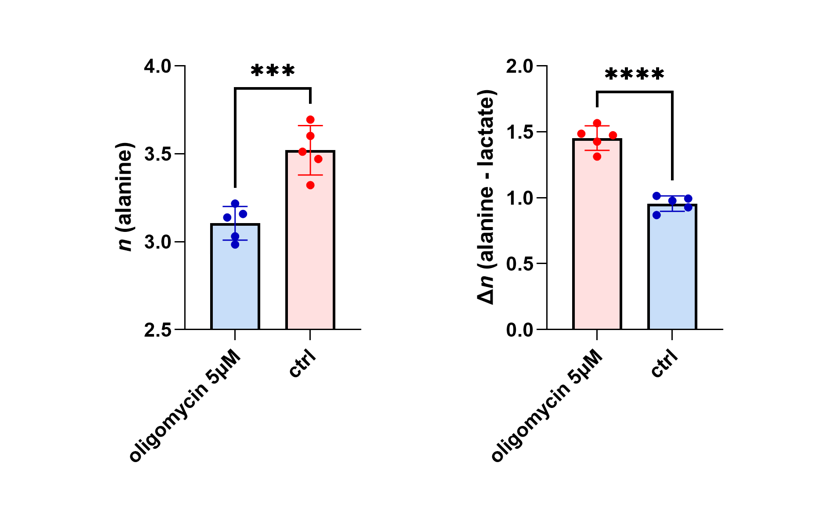


**Supporting Figure 1:** Extension of data presented in Figure 2. Alanine *n* for oligomycin treated HepG2 cells is 3.10 ± 0.096 and in the basal condition 3.52 ± 0. 14 (*p* = 6.02E-4). The difference between alanine *n* and lactate *n* in oligomycin-treated cells is 1.45 ± 0.093 and in the basal condition 0.95 ± 0.058 (*p* < 0.0001).


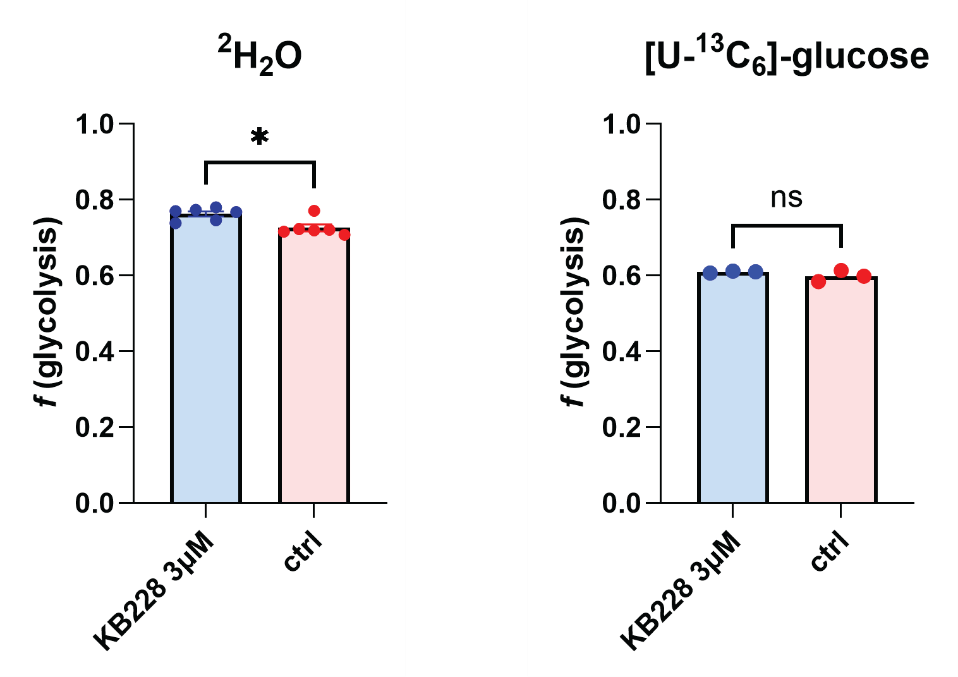


**Supporting Figure 2:** Treatment of HepG2 with glycogen phosphorylase inhibitor KB228 with ^2^H_2_O labeling yields *f* (glycolysis) of 76.1 ± 1.77% and in the basal condition is 72.5 ± 2.3% (*p* = 0.01) and with [U-^13^C_6_]-glucose labeling yields 60.9 ± 0.26% versus 59.9 ± 1.4%, respectively (*p =* 0.284)

All the supplementary information tutorial files mentioned in the manuscript are found here:

<https://www.dropbox.com/scl/fo/3xfsxghnyjm2k4kdgo0tp/AO8FUNDDBHh6ca1e6pb9QvQ?rlkey=8aek05tmt36bwl6zn50apw5p2&st=cpvjvp4u&dl=0>
